# Triclosan Potentiates Epithelial-To-Mesenchymal Transition in Anoikis-Resistant Human Lung Cancer Cells

**DOI:** 10.1371/journal.pone.0110851

**Published:** 2014-10-16

**Authors:** Thidarat Winitthana, Somsong Lawanprasert, Pithi Chanvorachote

**Affiliations:** 1 Department of Pharmacology and Physiology, Faculty of Pharmaceutical Sciences, Chulalongkorn University, Bangkok, Thailand; 2 Cell-Based Drug and Health Product Development Research Unit, Faculty of Pharmaceutical Sciences, Chulalongkorn University, Bangkok, Thailand; Wayne State University School of Medicine, United States of America

## Abstract

Alteration of cancer cell toward mesenchymal phenotype has been shown to potentiate tumor aggressiveness by increasing cancer cell metastasis. Herein, we report the effect of triclosan, a widely used antibacterial agent found in many daily products, in enhancing the epithelial-to-mesenchymal transition (EMT) in aggressive anoikis resistant human H460 lung cancer cells. EMT has been long known to increase abilities of the cells to increase migration, invasion, and survival in circulating system. The present study reveals that treatment of the cancer cells with triclosan at the physiologically related concentrations significantly increased the colony number of the cancer cells assessed by tumor formation assay. Also, the mesenchymal-like morphology and decrease in cell-to-cell adhesion were observed in triclosan-treated cells. Importantly, western blot analysis revealed that triclosan-treated cells exhibited decreased E-cadherin, while the levels of EMT markers, namely N-cadherin, vimentin, snail and slug were found to be significantly up-regulated. Furthermore, EMT induced by triclosan treatment was accompanied by the activation of focal adhesion kinase/ATP dependent tyrosine kinase (FAK/Akt) and Ras-related C3 botulinum toxin substrate 1 (Rac1), which enhanced the ability of the cells to migrate and invade. In conclusion, we demonstrated for the first time that triclosan may potentiate cancer cells survival in detached condition and motility via the process of EMT. As mentioned capabilities are required for success in metastasis, the present study provides the novel toxicological information and encourages the awareness of triclosan use in cancer patients.

## Introduction

**Figure 1:**
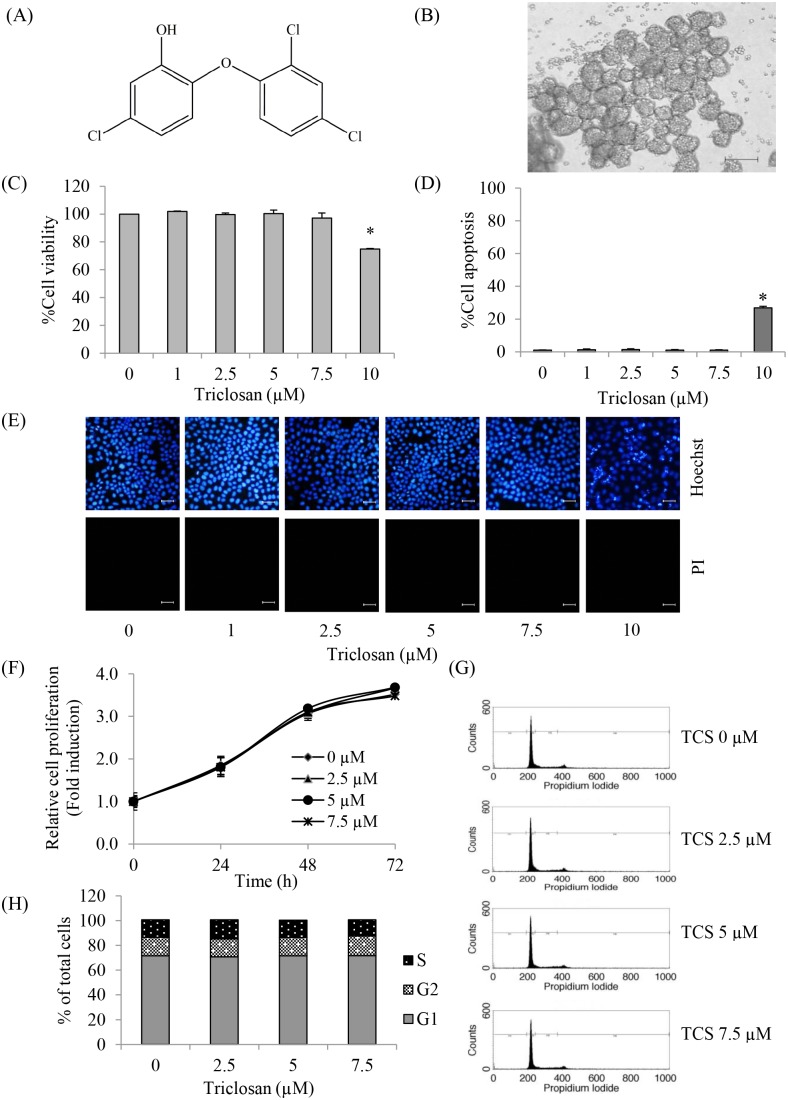
Cytotoxic effect and proliferative effect of TCS on anoikis resistant H460 cells. (A) The chemical structure of TCS. (B) Multicellular aggregation of anoikis resistant H460 cells. Scale bar is 1,000 µm. (C–D) After treatment with TCS (0–10 µM) for 24 h, the percentage of cell viability was determined by MTT assay and the percentage of apoptotic cells was detected by Hoechst33342 staining, respectively. Values are means of the three independent triplicate samples ± SE. *P<0.05 versus non-treated control. (E) After the indicated treatment, nuclear morphology of the cells was detected by Hoechst33342/PI co-staining assay and visualized under a fluorescence microscope. Scale bar is 50 µm. (F) Cells were treated with TCS (0–7.5 µM) for 24, 48 or 72 h. Cell viability was determined by MTT assay. The data represent the means of the three independent triplicate samples ± SE. *P<0.05 versus non-treated control at each indicated time. (G–H) Cells were treated with TCS (0–7.5 µM) for 48 h. Cell cycle of TCS-treated cells was determined by PI staining and flow cytometry. *P<0.05 versus non-treated control.

The well-known broad-spectrum anti-bacterial agent triclosan (2,4,4′ –trichloro-2′-hydroxydiphenyl ether; TCS) (Figure 1A) has been commercially used in a variety of products to inhibit the growth of bacteria, fungi, and mildew [1], [2]. TCS has been used under the regulation of the Food and Drug Administration (in cosmetics, deodorant, hand soaps, toothpaste) as well as the Environmental Protection Agency (in materials preservative incorporated into household plastics and textiles) [2], [3]. The concentrations used of TCS in different products may vary; however, its levels in most personal care products range from 0.1–2% [1], [3]. The fact that the significant levels of TCS are detectable in the plasma of TCS-exposed human at the concentration ranging from 0.02 and 20 µg/ml (0.069 and 69 µM) leads to the possible conception that this agent may possibly impact human physiology [4].

Focusing on cancer, up-to-date information has pointed out that TCS has insignificant effects on carcinogenesis and direct gene mutation [2], [5], [6]. However, considering that TCS is a substance that people can be exposed to for a long period in their life, it is important to fully understand the possible effects of this agent not only on carcinogenesis but also the possible impact on cancer cell behaviors. Recent studies have indicated that the transition of cellular phenotype from epithelial to mesenchymal named epithelial-to-mesenchymal transition (EMT) is a critical factor in facilitating metastasis of many cancers [7]–[9]. EMT has received considerable attention in cancer-related researches and EMT has been recognized as a hallmark of cancer stemness as well as aggressiveness [10]. EMT process has resulted in the alteration of cell behaviors which, in most cases, enhances ability to metastasize, including potentiated migration of the cells from its primary tumor, and increased resistance to apoptosis [11]–[13].

Most evidence has suggested that the sub population of cancer cells that exhibit anoikis resistant property is the majority of cells undergoing successful metastasis [14]–[18]. Anoikis resistant cells are also known as circulating tumor cells (CTCs) [19]. In clinical practice, CTCs have been considered to be a potential biomarker that reflects cancer aggressiveness of many types of cancer including breast, prostate, colorectal, bladder, gastric, liver and lung cancers [20]–[24]. The presence and quantity of CTCs in peripheral blood are shown to correlate well with poor prognosis in cancer patients [19], [20]. The population of CTCs exhibit heterogeneous cell phenotypes including epithelial, mesenchymal, and those phenotypes in a transitional state from epithelial to mesenchymal [20], [24]–[27]. As the process of EMT resulted in the mesenchymal phenotypes with increase metastasis potencies including anoikis resistance and invasive ability of cells [14], [20], [23] factors or stimuli that facilitate this EMT in CTCs may alter the phenotypes of CTCs population and affect the metastasis potentials of the cells. As CTCs are found in systemic circulation [19], [24], [28], the cells are likely to be exposed to several chemicals existing in the blood. Based on such a concern, several compounds have been investigated and reported to have an EMT-inducing property such as TGF-β [29], [30], epidermal growth factor [31], [32], celecoxib [33] gefitinib [34] and hexavalent chromium [35].

Although the presence of certain concentrations of TCS has been reported in human circulations, the information regarding effects of such an agent on EMT process of CTCs is still largely unknown. The present study aims to investigate the effects as well as the possible effects of this compound on the aggressive population of lung cancer cells. Better understandings obtained from this study may contribute to the safer use of TCS and provide new assessment approaches for cancer-related toxicity.

## Materials and Methods

### 1. Cells and reagents

NCI-H460 was obtained from the American Type Culture Collection (ATCC, Manassas, VA, USA). The cancer cells were cultured in RPMI-1640 medium supplemented with 10% fetal bovine serum (FBS), 2 mM L-glutamine, 100 IU/ml penicillin, and 100 µg/ml streptomycin (Life technologies, MD, USA) in 37°C with 5% CO_2_ humidified incubator. Triclosan was obtained from Sigma (St. Louis, MO, USA). TCS was diluted with sterile medium to achieve the working concentrations with 0.1% DMSO in the final solution. As regards the sources of reagents, Hoechst33342, propidium iodide (PI), phalloidin tetramethylrhodamine B isothiocyanate, bovine serum albumin (BSA) and dimethylsulfoxide (DMSO) were purchased from Sigma (St. Louis, MO, USA). 3-(4,5-Dimethylthiazol-2-yl)-2,5-diphenyltetrazolium bromide (MTT) were purchased from Gibco (Life technologies, MD, USA). Matrigel was obtained from BD Biosciences, Inc. (Woburn, MA, USA). BCA assay kit and Supersignal west pico chemiluminescent was obtained from Thermo Scientific, Inc. (Rockford, IL, USA). Rabbit monoclonal antibodies for E-cadherin, N-cadherin, vimentin, slug, snail, Akt, phosphorylated Akt (S473), focal adhesion kinase (FAK), phosphorylated FAK (Y397), β-actin, peroxidase conjugated anti-rabbit IgG and peroxidase conjugated anti-mouse IgG were obtained from Cell Signaling (Denvers, MA, USA). Mouse monoclonal antibodies for Active Rac1-GTP and Active Rho-GTP were obtained from NewEast Biosciences (Malvern, PA, USA). Immobilon Western chemiluminescent HRP substrate was obtained from Millipore, Corp (Billerica, MA, USA) and Thermo Fisher Scientific Inc. (Rockford, IL, USA).

### 2. Anoikis resistant cells

Anoikis resistant cell culture was carried out according to method of Sakuma et al. [36] and Khongmanee et al. [37] with minor modifications. In brief, attached H460 cells were trypsinized when cells reached 80–90% confluence with 0.05% trypsin/0.02% EDTA. Then cells were cultured in ultralow attachment 6-well plate (Corning Costar, MA, USA) in RPMI-1640 medium while other conditions as described for attachment culture were maintained. Cells were cultured at a density of 2×10^5^ cells/ml for 48 h. Suspended cells were then collected and prepared into a single cell suspension by 1 mM EDTA treatment. Then cells were washed with complete RPMI-1640 medium. Cell viability was measured using automated cell counter. Viable cells were used for further experiments.

### 3. Cell viability assay and cell proliferation assay

Cells viability was determined by MTT assay. Cells were seeded at a density of 1×10^4^ cells/well onto 96-well plate overnight. After that they were treated with various concentrations of TCS for 24 h. Following the treatment, the medium was then replaced with MTT solution (5.0 mg/ml in PBS) and incubated at 37°C for 4 h. Then the medium was replaced with 100 µl DMSO to solubilize the formazan product and the intensity of the formazan product was measured at 570 nm using a microplate reader (Anthros, Durham, NC, USA). Cell viability was expressed as the percentage calculated from the optical density of treated cells relative to the controlled cells. Meanwhile, cell proliferative effect was determined also using MTT assay. Cells were seeded at a density of 2×10^3^ cells/well in 96-well plate and incubated overnight. After that, the cells were treated with various concentrations of TCS for 0, 24, 48, and 72 h. Cell proliferation was measured by MTT assay as described in cell viability assessment.

### 4. Nuclear staining assay

Apoptotic and necrotic cell death were determined by Hoechst33342 and PI co-staining. Cells were seeded at a density of 1×10^4^ cells/well onto 96-well plate and incubated overnight. Cells were then treated with TCS for 24 h. After specific treatments, cells were incubated with 10 µg/ml of Hoechst33342 and 5 µg/ml of PI for 30 min at 37°C. The apoptotic cells having condensed chromatin and/or fragmented nuclei and PI-positive necrotic cells were visualized and scored under a fluorescence microscope (Olympus IX51 with DP70, Olypus America Inc., Center valley, PA, USA).

### 5. Cell cycle analysis

After the treatment of the cells with TCS for 48 h, the cells were trypsinized and fixed with 70% absolute ethanol at −20°C overnight. the cells were then washed with cold PBS and incubated in PI solution containing 0.1% Triton-X, 1 µg/ml RNase, and 1 mg/ml propidium iodide at 37°C for 30 min. DNA in whole cells were stained with PI and cell cycle profile was analyzed using flow cytometry (FACSort, Becton Dickinson, Rutherford, NJ, USA).

### 6. Colony formation assay

Upon treatment with TCS, anchorage-independent growth was examined via colony formation assay in accordance with the method of Koleske et al. [38] with minor modifications. Briefly, cells were treated with TCS at non-toxic concentrations for 24 h and then treated with 1 mM EDTA to prepare single cell suspension. The cells were suspended in RPMI-1640 containing 10% FBS and 0.33% agarose, then 250 µl containing 1×10^3^ cells were embedded as a second layer in a 24-well plate over a 500 µl base layer containing 10% FBS and 0.5% agarose. The cells were fed every 3 days by adding 250 µl of complete medium. After 7 and 10 days, the resulting colonies were photographed at ×4 magnification. Colony number and colony size were determined on the 10^th^ day of culture.

### 7. Filopodia characterization

Filopodia was characterized by phalloidin-rhodamine staining assay as described in Kowitdamrong et al. [39]. Cells were treated with TCS at non-toxic concentrations for 24 h in detach condition and seeded at a density of 2×10^3^ cells/well onto 96-well plate for 4 h. The cells were then washed with PBS, fixed with 4% paraformaldehyde in PBS for 10 min at 37°C, permeabilized with 0.1% Triton-X100 in PBS for 4 min, and blocked with 0.2% BSA for 30 min. Following that, the cells were incubated with 1∶100 phalloidin-rhodamine in PBS for 15 min and washed with PBS 3 times. Filopodia was then imaged by a fluorescence microscope (Olympus IX51 with DP70).

### 8. Migration assay

Migration was determined by Boyden chamber assay as previously as described in Kowitdamrong et al. [39]. Cells were pretreated with TCS at non-toxic concentrations for 24 h in detached condition. Then the cells were seeded at a density of 5×10^4^ cells/well onto an upper 24-transwell plate of the transwell filter (8-µM pore) in the medium containing 0.1% serum and 500 µl of complete medium was added to the lower chamber. After 24 h, the non-migrated cells in the upperside membrane were removed by cotton-swab wiping. The cells that migrated to the underside of the membrane were stained with 10 µg/ml Hoechst33342 for 30 min. The cells were then visualized and scored under a fluorescence microscope (Olympus IX51 with DP70).

### 9. Invasion assay

The invasion assay was carried out using 24-transwell chambers as previously as described in Kowitdamrong et al. [39]. Transwells were coated with 50 µl of 0.5% matrigel on the upper surface of chamber and incubated overnight at 37°C in a humidified incubator. After treatment with TCS at non-toxic concentrations for 24 h in detached condition, cells were seeded at a density of 5×10^4^ cells/well onto the upper chamber in medium containing 0.1% serum and 500 µl of complete medium was added to the lower chamber. After 24 h, the non-invaded cells in the upperside of membrane were removed by cotton-swab wiping. Invaded cells at the basolateral side of membrane were fixed with cold absolute methanol for 10 min and stained with 10 µg/mL Hoechst33342 for 30 min. Cells were then visualized and scored under a fluorescence microscope (Olympus IX51 with DP70).

### 10. Western blot analysis

After specific treatments, cells were incubated in a lysis buffer containing 20 mM Tris–HCl (pH 7.5), 1.5% Triton X-100, 150 mM sodium chloride, 10% glycerol, 1 mM sodium orthovanadate, 50 mM sodium fluoride, 1 mM phenylmethylsulfonyl fluoride, and a commercial protease inhibitor mixture (Roche Applied Science, Indianapolis, IN, USA) for 2 h on ice. Cell lysates were collected and protein content was determined using the Bradford method (Bio-Rad Laboratories, Hercules, CA). Equal amounts of protein from each sample were denatured by heating at 95°C for 5 min with loading buffer and subsequently loaded onto a 7.5% SDS-polyacrylamide gel electrophoresis for the detection of EMT markers and E-cadherin expression or 10% SDS-polyacrylamide gel electrophoresis for the detection of migratory-related protein expression. After separation, the proteins were transferred onto 0.45 µM nitrocellulose membranes. The transferred membranes were blocked in 5% non-fat dry milk in TBST (25 mM Tris-HCL (pH 7.5), 125 mM NaCl, 0.05% Tween 20) for 1 h, and then incubated with a specific primary antibody (1∶1,000 dilution) overnight at 4°C. The membranes were washed three times with TBST for 5 min and incubated with Horseradish peroxidase-coupled isotype-specific secondary antibodies (1∶2,000 dilution) for 2 h at room temperature. The membranes were washed three times with TBST for 5 min and the immune complexes were detected by enhancement with a chemiluminescent substrate and quantified using analyst/PC densitometry software (Bio-Rad Laboratories, Hercules, CA, USA).

### 11. Statistical analysis

Data were obtained from three independent experiments and presented as means ± standard error (SE). Statistical analyses were performed using one-way ANOVA and post hoc test (Turkey’s test) at a significance level of *P*-values<0.05. SPSS 17.0 was used for all statistical analyses.

## Results

### 1. Effects of TCS on anoikis resistant H460 cells

Anoikis resistant cancer cells were used as a model for studying CTCs [36], [37], [40]. Anoikis resistant lung cancer cells were generated as described in Materials and Methods. It was found that detached H460 cells spontaneously formed multicellular aggregates after culture for 48 h (Figure 1B). To elucidate the possible effect of TCS on CTC lung cancer cells, cytotoxic effect of the compound on the cells was first characterized. TCS at the concentrations ranging from 0.069 and 69 µM was found in the plasma of TCS-exposed subjects [4]. Therefore, the anoikis resistant cells were incubated with TCS at the concentrations of 0–10 µM for 24 h and cell viability was assessed by MTT assay. Figure 1C shows that TCS treatment significantly decreased cell survival at the dose of 10 µM with approximately 80% of cells remaining viable, while treatment of the cells with TCS at 0–7.5 µM caused no significant toxic effect. Hoechst33342/PI staining assay confirmed that apoptosis and necrosis were not detectable in the TCS-treated cells at 0–7.5 µM. The apoptotic cells with fragmented or condensed nuclei were only detected in the cells treated with 10 µM TCS (Figures 1D and E). Therefore, the concentrations of TCS at 0–7.5 µM were used for following experiments.

Having shown the toxic effect of TCS on anoikis resistant cells, we next investigated the effects of TCS on cell proliferation and cell cycle. Cells were treated with non-toxic concentrations of TCS for 0–72 h and proliferation of the cells was determined as described in Materials and Methods. Proliferation response of anoikis resistant cells to TCS was shown in Figure 1F. TCS-treated cells showed no significant difference in terms of cell proliferation in comparison to the non-treated control cells. These results were confirmed by cell cycle analysis using PI and flow cytometry. The results indicated that TCS treatment caused no significant effects on the cell cycle (Figures 1G and 1 H). Together, the results suggested that TCS possessed no proliferative effect on anoikis resistant H460 cells in normal culturing condition.

### 2. TCS promoting cell growth in anchorage–independent manner

**Figure 2:**
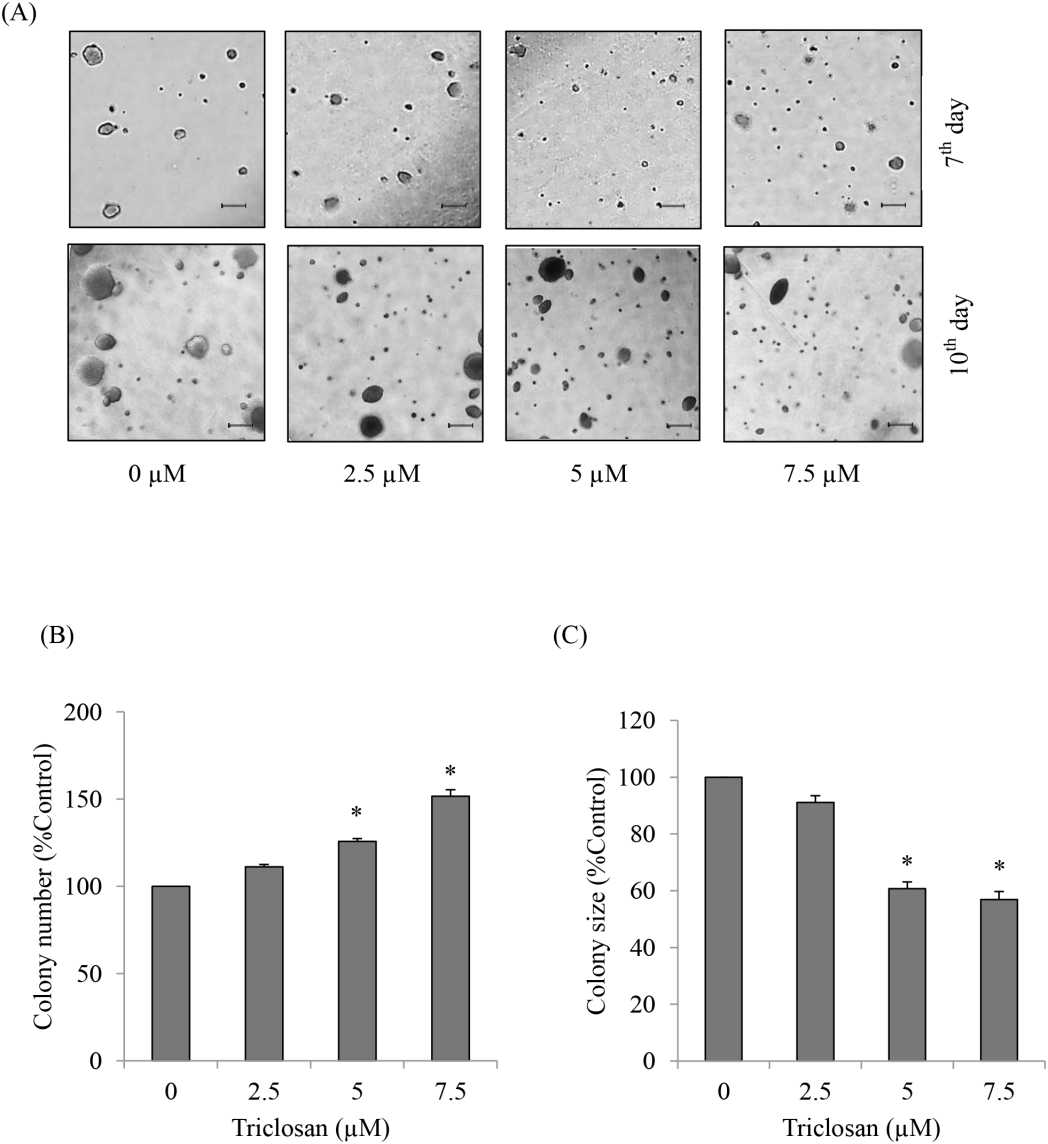
Effects of TCS on anchorage-independent growth of anoikis resistant H460 cells. (A) Cells were pretreated with TCS (0–7.5 µM) for 24 h and subjected to soft agar colony formation assay, as described in “Materials and Methods”. Representative fields from three independent experiments were photographed after the cells were cultured for 7 and 10 days. Scale bar is 1,000 µm. (B–C) Colony number and colony size were determined by image analyzer on the 10^th^ day of culture. Values are means of the three independent triplicate samples ± SE. *P<0.05 versus non-treated control.

Because anchorage-independent growth of the cancer cells has been shown to augment metastasis, we next investigated the effect of TCS on cancer cell growth in such condition. In doing this, anoikis resistant cells H460 were treated with TCS for 24 h before they were subjected to colony formation assay. The cells were then seeded in agarose layer to prevent cell-cell interaction and attachment. Colony number and colony size were obtained by photographing and counting after the cells were cultured for 7 and 10 days. The colony formation was shown in Figure 2A. Colony number and colony size of each treatment were calculated as a percentage of the control group and shown in Figure 2B and C, respectively. The results indicated that TCS at the concentrations of 5 and 7.5 µM significantly increased colony formation of anoikis resistant H460 cells. However, TCS at both concentrations significantly reduced colony size in comparison to that of non-treated control group. Such observations indicated that TCS promoted anchorage-independent survival of the cells, but decreased the growth rate of the cells in detached condition. Our results consists with the previous findings that the increase in anchorage-independent survival with low proliferative ability has been observed in the cancer cells undergoing EMT [11], [12], [41], [42].

### 3. TCS inhibiting cell-cell interaction

**Figure 3:**
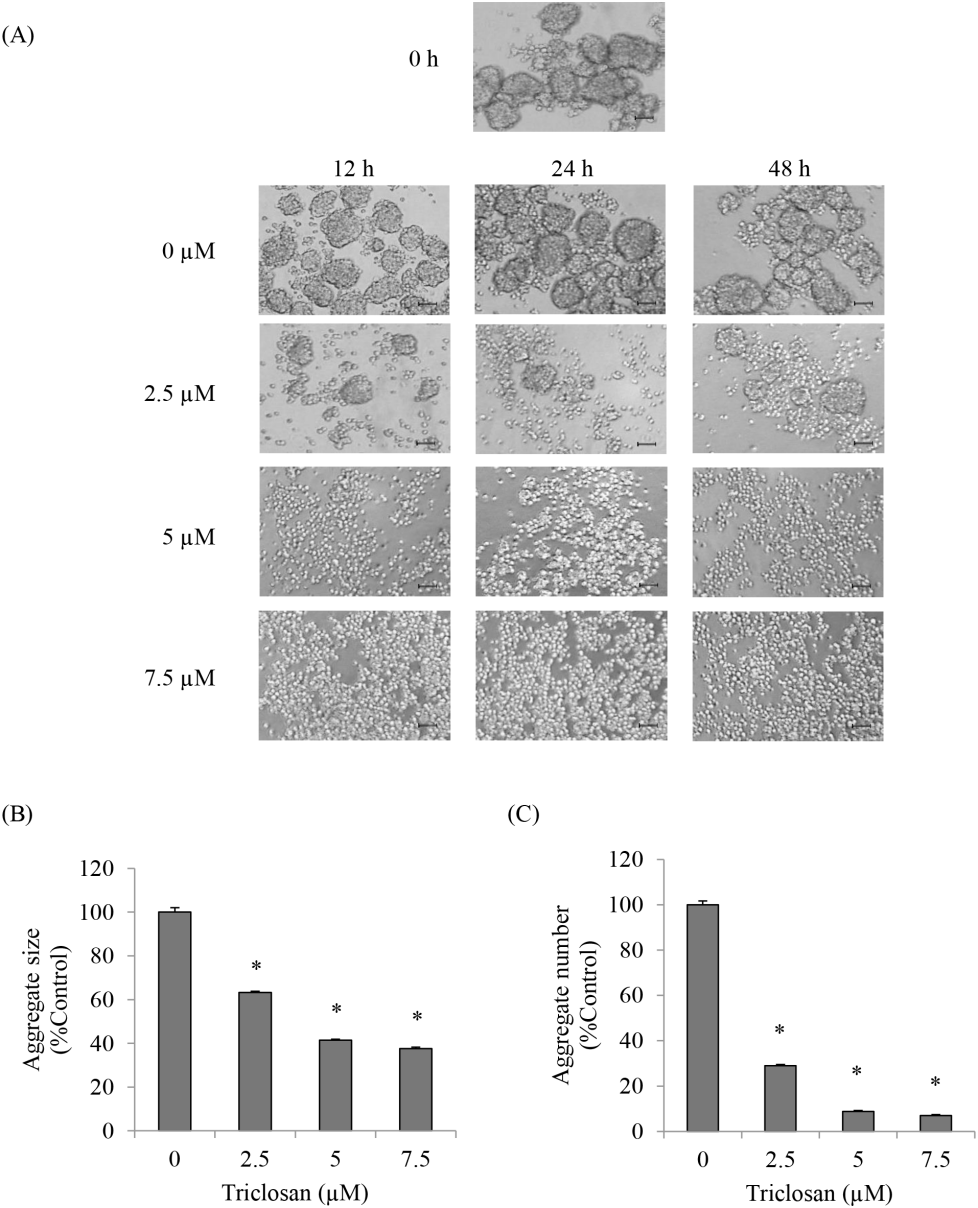
The effects of TCS on cell-cell interaction of anoikis resistant H460 cells. (A) Cells were treated with TCS (0–7.5 µM) for 12, 24 or 48 h in detached condition and cell-cell interaction was photographed. Scale bar is 1,000 µm. (B–C) After the treatment with TCS (0–7.5 µM) for 24 h in detached condition, aggregate size and aggregate number were determined by image analyzer. The data present means of the three independent triplicate samples ± SE. *P<0.05 versus non-treated control.

Loss of cell-cell adhesion was found in the cells during the process of EMT as a results from cadherin switching [7], [8]. To examine the effect of TCS on cell-cell adhesion of anoikis resistant H460 cells, we seeded cells in 24-well low attach plate at the density of 1.5×10^5^ cells/well and treated them with non-toxic concentrations of TCS. The cell-cell interaction was observed in formation of cell aggregation and photographed using a phase-contrast microscope. Aggregate size and number were determined and calculated relative to the non-treated control. Figure 3A shows that TCS treatment significantly altered the aggregate behavior of the cells to single cell suspension. Addition of TCS resulted in the significant reduction of both number and size of multi-cellular aggregates in a dose-dependent manner (Figure 3B and C). These results suggested that TCS promoted loss of cell-cell adhesion of anoikis resistant H460 cells which is a dominant characteristic of the cells undergoing EMT.

### 4. TCS increasing the expression of EMT markers

**Figure 4:**
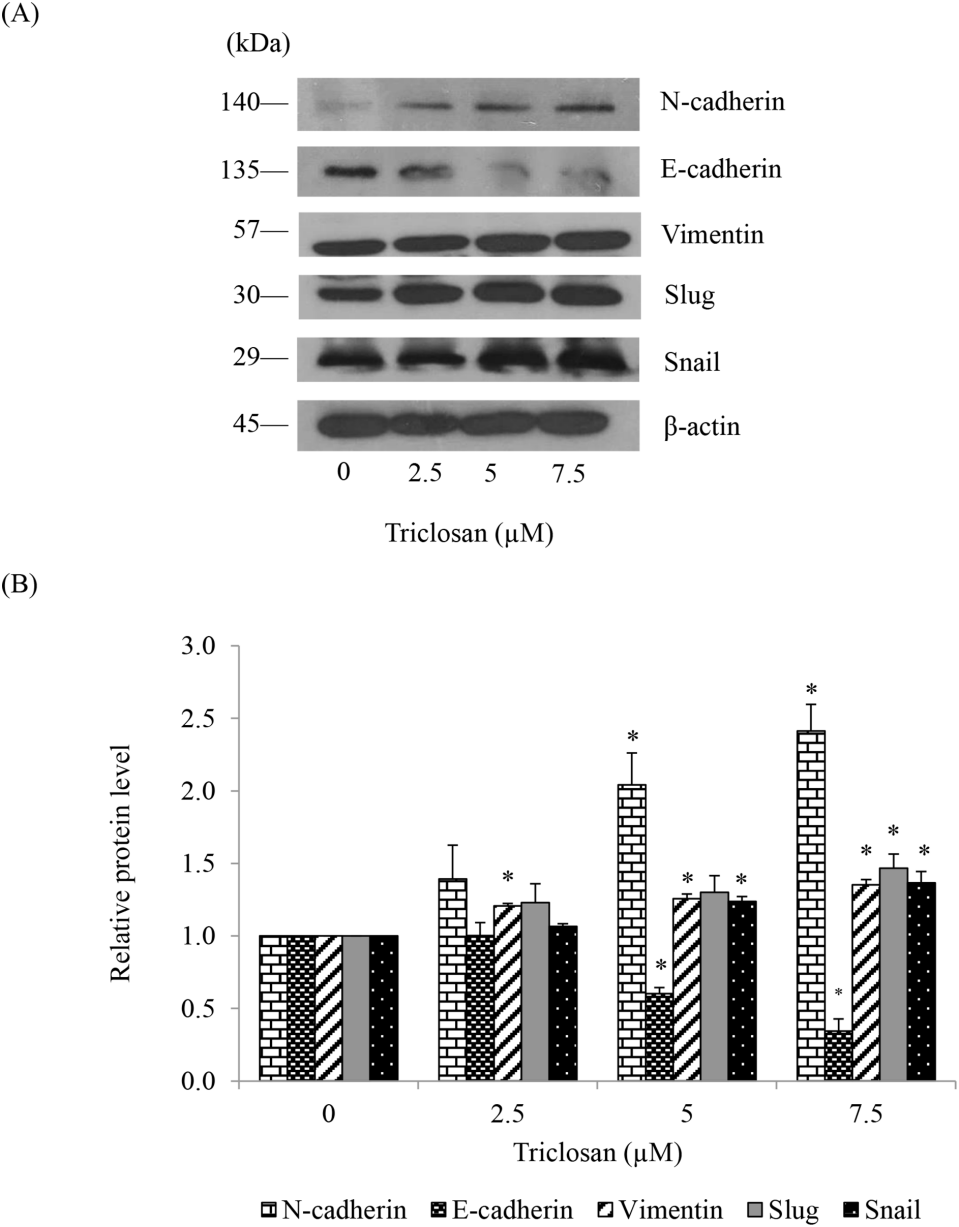
Effects of TCS on E-cadherin and EMT markers. (A) Anoikis resistant H460 cells were treated with TCS (0–7.5 µM) for 24 h in detached condition. The level of N-cadherin, E-cadherin, vimentin, slug and snail were determined by western blotting. Blots were reprobed with β-actin to confirm equal loading. (B) The immunoblot signals were quantified by densitometry and mean data from independent experiments were normalized to the results. The data present means of the three independent triplicate samples ± SE. *P<0.05 versus non-treated control.

We next investigated the effect of TCS treatment on EMT markers including N-cadherin, vimentin, snail, and slug. Anoikis resistant H460 cells were treated with non-toxic concentrations of TCS for 24 h and EMT markers were evaluated by western blotting. Figures 4A and B show that the expression level of E-cadherin was significantly decreased in response to TCS treatment in a concentration-dependent manner. In addition, TCS significantly enhanced the increase of N-cadherin, vimentin, slug, and snail. The expression of snail was significantly enhanced by TCS treatment at 5 and 7.5 µM in a dose-dependent manner, while the expression of slug was only significantly induced by the treatment with TCS at 7.5 µM. These results suggested that TCS increased EMT phenotypes in these cells.

### 5. TCS-mediated EMT enhancing cells migration and invasion

**Figure 5:**
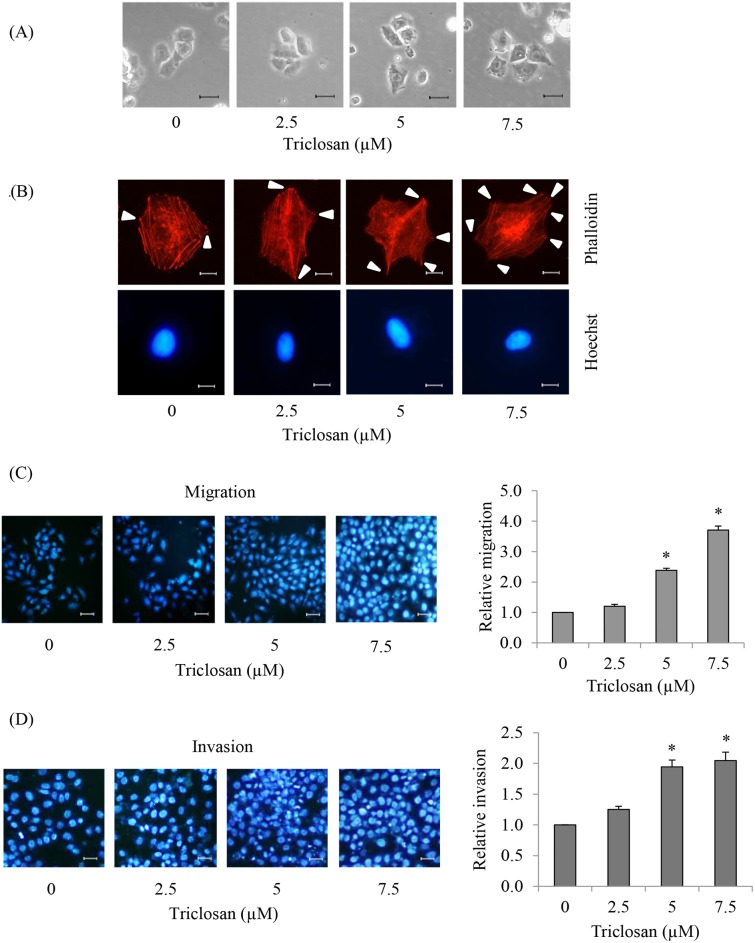
Effects of TCS-mediated EMT on migratory and invasive ability of anoikis resistant H460 cells. (A) Cells were treated with TCS at 0–7.5 µM for 24 h and then attached on conventional culture dishes for 4 h. Cell morphology was detected by phase contrast microscopy. Scale bar is 25 µm. (B) After the indicated treatment, filopodia and viable cells were detected by phalloidin-rhodamine or Hoechst33342 staining, respectively. Cells were visualized under fluorescence microscope. Filopodia protrusions of each treatment were indicated by arrows. Scale bar is 5 µm. (C) Cells were pretreated with TCS (0–7.5 µM) for 24 h. Transwell assay was used to investigate cell migration. Migratory cells at the basolateral side of membrane were stained with Hoechst33342 and visualized under fluorescence microscopy. Scale bar is 50 µm. The average numbers of migratory cells in each field at the basolateral side of membrane were plotted relative to the control group. Values are means of the three independent triplicate samples ± SE. *P<0.05 versus non-treated control. (D) After treatment with TCS (0–7.5 µM) for 24 h, cell invasion was evaluated using transwell coated with matrigel as described in “Materials and Methods”. Invaded cells at the basolateral side of membrane were stained with Hoechst33342 and visualized under fluorescence microscopy. Scale bar is 50 µm. The average numbers of invaded cells in each field across the membrane were plotted relative to control group. Values are means of the three independent triplicate samples ± SE. *P<0.05 versus non-treated control.

An important hallmark of aggressive cancer cells is their high ability to migrate and invade [43], [44]. EMT is recognized as an important factor facilitating cell motility [12], [45]. Cells were treated with TCS at non-toxic concentrations for 24 h and the migration and invasion behaviors of the cells were determined as described in Materials and Methods. Figure 5A indicates that TCS-treated cells exhibited increased polarity and filopodia. Filopodia of the TCS-treated cells was stained with phalloidin-rhodamine as shown in Figure 5B. TCS-treated cells exhibited filopodia protrusions accumulating at the border of cells in a dose-dependent fashion. Additionally, migration and invasion assay revealed that TCS increased cell migration and invasion (Figures 5C and D). The above findings suggested that EMT induction upon TCS treatment promoted filopodia formation and potentiated migratory and invasive abilities of anoikis resistant H460 cells.

**Figure 6:**
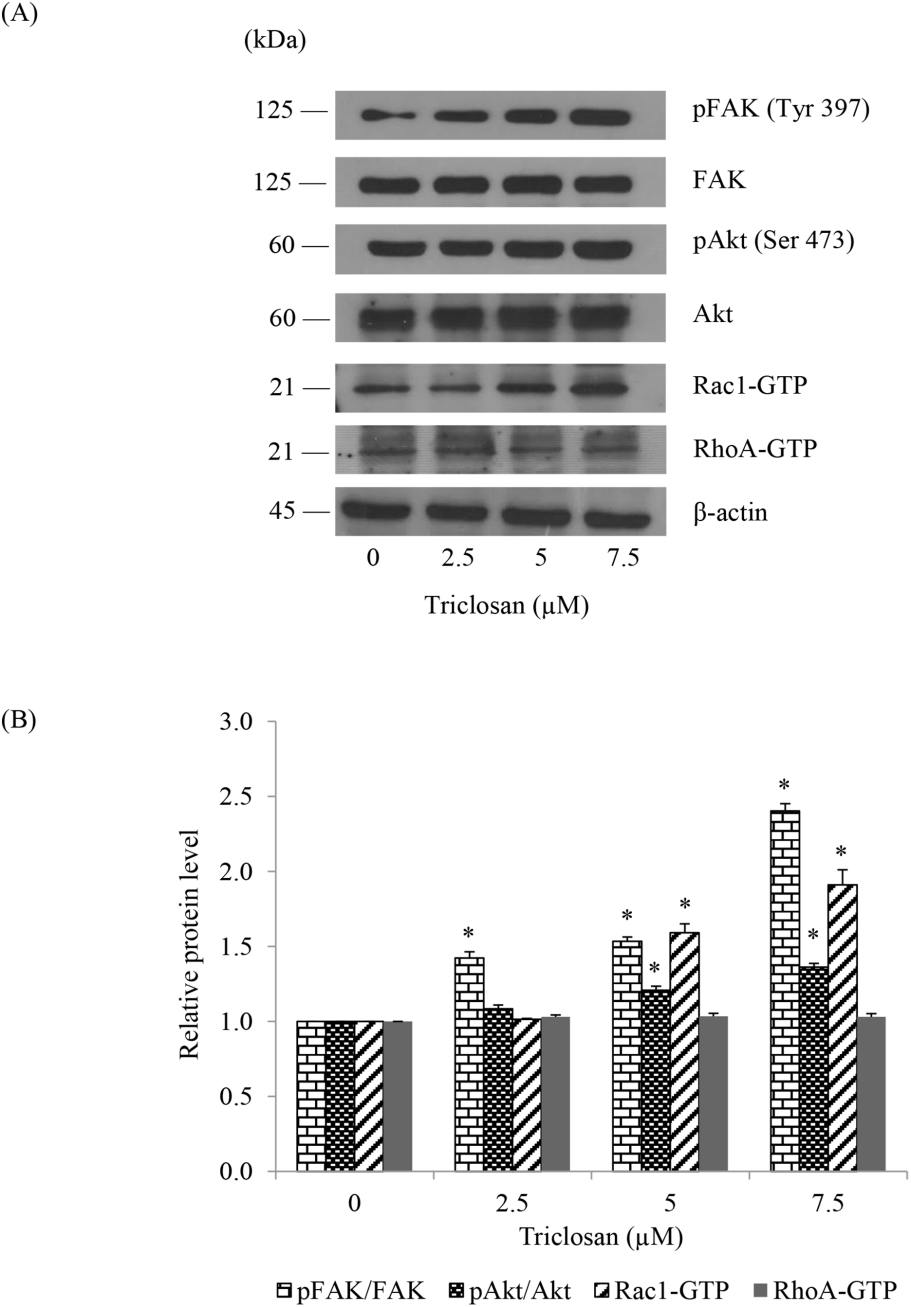
Effects of TCS-mediated EMT on migratory-related proteins. (A) Anoikis resistant H460 cells were treated with TCS at 0–7.5 µM for 24 h in detached condition and then attached on conventional culture dishes for 4 h. The level of pFAK (Tyr 397), FAK, pAkt (Ser 473), Akt, activated Rac1 (Rac1-GTP) and activated RhoA (RhoA-GTP) were determined by western blotting. Blots were reprobed with β-actin to confirm equal loading. (B) The immunoblot signals were quantified by densitometry and mean data from independent experiments were normalized to the results. Values are means of the three triplicate independent samples ± SE. *P<0.05 versus non-treated control.

Furthermore, the down-stream effector proteins which are responsible for cell motility were determined using western blotting. The cells were treated with indicated concentrations of TCS for 24 h and subjected to western blot analysis. The expression levels of migratory-related proteins including activated FAK (phosphorylated FAK, Tyr 397), FAK, activated Akt (phosphorylated Akt, Ser 473), Akt, activated Rac1 (Rac1-GTP), and activated RhoA (RhoA-GTP) were investigated. Figures 6A and B show that TCS treatment significantly increased the level of phosphorylated FAK, activated Akt, and active Rac1-GTP. However, TCS possessed no significant effect on activated RhoA level. These results suggested that TCS-induced EMT promoted the motility of anoikis resistant H460 cells through the activation of FAK/Akt signaling pathway as well as Rac1 activation.

## Discussion

Lung cancer has garnered most attentions in cancer research field since this type of cancers is considered as a major cause of cancer-related mortality [46]. Indeed, the high metastasis rate in such a cancer makes it the most life-threatening and in most cases metastasis is detected at the time of first diagnosis [47]. Because the ability of cancer cells to metastasize can be augmented by the process of EMT [9], [12], [48], the present study reporting the positive regulatory effect of TCS on the EMT of human cancer cells highlights the possible novel toxicity caused by this compound.

TCS has been widely used for over 30 years in health care products as well as in medical devices. TCS is readily and completely absorbed following oral administration. TCS has been identified in urine, plasma, and breast milk of humans with a wide range of levels depending upon the individual’s daily intake, its the concentration in the products as well as the route and frequency of administration [2], [3]. The low and high concentrations of TCS in human plasma have been reported at 0.02 and 20 µg/ml (0.069 and 69 µM) respectively [4]. Previous studies reported the possible effects of TCS in the induction of hepatocellular adenoma and carcinoma formations in rodent model [2]. Recently, Ma et al. (2013) found that TCS reduced global DNA methylation (GDM) in HepG2 cells and they proposed the reduction as the possible mechanism of TCS promoting tumor in rodents [49]. Since global DNA hypomethylation is associated with aberrant gene expression, loss of imprinting, chromosome instability and anomalies, it has been shown to play a key role in controlling EMT and cancer metastasis [50]. Thus, TCS induced global DNA hypomethylation raises a question whether TCS can promote cancer metastasis in the carcinogenesis safety aspect of this compound.

Anoikis resistant H460 cells were found to form multicellular aggregation in suspended condition and still express a high level of E-cadherin which is consistent with previous studies [36], [40]. Together with this finding, the sub-population of CTCs was clinically found as multicellular aggregates with epithelial marker expression [25], [51]. The transition of the cells towards mesenchymal phenotypes through EMT has been shown to enhance the ability of CTCs cells to be more aggressive in many cancers [14], [20]. In this study, we employed anoikis resistant H460 cells as a model of CTCs and found that TCS enhanced EMT of such cells and promoted the ability of the cells to migrate and invade.

Our results indicated that TCS treatments significantly increased the cells’ ability to survive in anchorage-independent condition (Figure 2) while decreased the proliferation rate of the cells. Such results were consistent with the previous study reporting that the cancer cells undergoing EMT possess the ability to survive in detached condition with decreased rate of growth [11], [12], [41], [42]. The possible explanation on this phenomenon is that EMT-related transcription factors are responsible for the attenuation of cell proliferation through the impairment of cell cycle progression [41], [42]. Cadherin switching from E-cadherin to N-cadherin is considered as the hallmark event of EMT [7], [48], [52]. Also, mesenchymal phenotypes of cells can be confirmed by the increased expression of vimentin which is a type-III intermediate filament highly expressed in mesenchymal cells [15], [53] and the enhanced expression of snail and slug which are the transcription factors that regulate cadherin switching and that drive cells into EMT process [11], [52], [54], [55]. In the present study, we found that treatment of the anoikis resistant cells with TCS significantly mediated cadherin switch and increased vimentin, snail, and slug (Figure 4). Because TCS was shown to decrease the level of GDM in cancer cells [49] and global DNA hypomethylation was related to EMT and stem-like phenotype [50], the possible mechanism of TCS to induce snail and slug expression in this case may be, at least in part, due to such effects of TCS on global DNA hypomethylation.

Several studies have reported that FAK plays an important role in dynamic turnover of focal adhesion of cells, increases filopodia formation and also modulates cell migration. FAK activation was reported to lead to the phosphorylation of Akt, which resulted in cell movement [39], [56]. FAK/Akt phosphorylation also activated the GTP-binding of several Rho-family GTPases such as Rac1 and RhoA. Rac1 is essential to stimulate the formation of membrane ruffling, lamellipodia and focal-complex formation, while RhoA induces the formation of stress fibres and focal adhesion [56], [57]. We found that TCS-induced EMT significantly promoted FAK/Akt activation as well as Rac1 activation. However, TCS possessed no effect on the level of RhoA-GTP. These results were consistent with previous reports that N-cadherin and slug were able to activate Akt pathway involving in cell survival and migration [7], [58]. In addition, N-cadherin increased steady-state levels of activated Rac1, which resulted in the increase of actin remodeling [7]. Moreover when cells undergo EMT, free p120-catenin in cytoplasm was recruited to N-cadherin on cell surface, which resulted in the activation of Rac1 and the subsequent inhibition of RhoA activation [59].

In summary, our findings highlighted the effects of TCS in inducing EMT process in anoikis resistant human lung cancer cells. TCS-treated cells exhibited increased EMT phenotypes including increased anchorage-independent survival, low cellular proliferation and loss of cell-cell adhesion. Importantly, TCS promoted the cadherin switch and increased vimentin, snail, and slug. In addition, we found that such TCS-mediated EMT enhanced the ability of the cells to migrate and invade through FAK/Akt and Rac1-dependent mechanisms. Consequently, such findings show the potential effect of TCS in promoting EMT in CTCs which may result in cancer aggressiveness. Furthermore, this study provides some new toxicological information regarding TCS, which should lead to caution in the use of TCS in cancer patients.
